# Prophylactic veno-arterial extracorporeal membrane oxygenation in patients undergoing high-risk percutaneous coronary intervention

**DOI:** 10.1007/s12471-019-01350-8

**Published:** 2019-11-28

**Authors:** F. S. van den Brink, T. A. Meijers, S. H. Hofma, A. J. van Boven, A. Nap, A. Vonk, P. Symersky, K. D. Sjauw, P. Knaapen

**Affiliations:** 1 Department of Cardiology, Medisch Centrum Leeuwarden, Leeuwarden, The Netherlands; 2 Department of Cardiology, Location Vrije Universiteit Medisch Centrum, Amsterdam Universitair Medisch Centrum, Amsterdam, The Netherlands; 3 Department of Cardio-Thoracic Surgery, Location Vrije Universiteit Medisch Centrum, Amsterdam Universitair Medisch Centrum, Amsterdam, The Netherlands

**Keywords:** Veno-arterial extracorporeal membrane oxygenation, Percutaneous coronary intervention, High-risk percutaneous coronary intervention, Chronic total occlusion

## Abstract

**Purpose:**

Complex high-risk percutaneous coronary intervention (PCI) is challenging and frequently accompanied by haemodynamic instability. Veno-arterial extracorporeal membrane oxygenation (VA-ECMO) can provide cardiopulmonary support in high-risk PCI. However, the outcome is unclear.

**Methods:**

A two-centre, retrospective study was performed of all patients undergoing high-risk PCI and receiving VA-ECMO for cardiopulmonary support.

**Results:**

A total of 14 patients (92% male, median age 69 (53–83) years), of whom 50% had previous coronary artery disease in the form of a coronary artery bypass graft (36%) and a PCI (14%) underwent high-risk PCI and received VA-ECMO support. The main target lesion was a left main coronary artery in 78%, a left anterior descending artery in 14%, a right coronary artery in 7%, and 71% underwent multi-vessel PCI in addition to main target vessel PCI. The median SYNTAX score was 27.2 (8–42.5) and in 64% (9/14) there was a chronic total occlusion. Left ventricular function was mildly impaired in 7% (1/14), moderately impaired in 14% (2/14) and severely impaired in 64% (9/14). Cannulation was femoral-femoral in all patients. Median ECMO run was 2.57 h (1–4). Survival was 93% (13/14). One patient died during hospitalisation due to refractory cardiac failure. All other patients survived to discharge. Complications occurred in 14% (2/14), with one patient developing a transient ischaemic attack post-ECMO and one patient developing a thrombus in the femoral vein used for ECMO cannulation.

**Conclusion:**

VA-ECMO in high-risk PCI is feasible with a good outcome. It can be successfully used for cardiopulmonary support in selected patients.

## What’s new?

- Veno-arterial extracorporeal membrane oxygenation can be used for mechanical support in high-risk percutaneous coronary intervention.
- Outcome in regard to mortality and neurological outcome is good and the complication rate is low.
- Revascularisation can be achieved in 100% of cases.
- The procedure can be used without transfer to an intensive care unit.
- Future comparison with other support devices like the Impella will have to show which of these approaches is most favourable as regards outcome, revascularisation and patient comfort.

## Introduction

Patients with stable complex coronary artery disease, e.g. three-vessel disease, left main coronary artery disease or one or more chronic total occlusions (CTO), who need revascularisation can be treated with either percutaneous coronary intervention (PCI) or coronary artery bypass graft (CABG) [1]. Usually these cases are discussed by a heart team, whereby several risk stratification scores can be used to determine the risk of either form of revascularisation and a well-considered decision made about the most appropriate revascularisation strategy [1]. Examples of these scores are the SYNTAX I, SYNTAX II, EuroSCORE and for CTOs the J‑CTO (Japanese CTO Registry) score [1–4]. For many years, CABG was the treatment of choice for multi-vessel coronary artery disease, especially in diabetic patients [2, 5]. In the last decade or so, studies have shown that for non-complex multi-vessel coronary artery disease, CABG and PCI have equally favourable outcomes [3]. For complex multi-vessel disease, however, guidelines still advise the use of CABG rather than PCI [1].

Nonetheless, a considerable group of patients is not suitable for surgical revascularisation. This group comprises patients with advanced age, multiple comorbidities, poor left ventricular function, previous cardiothoracic surgery, or a combination of these factors. Until recently, treatment for these patients was either conservative or very high-risk PCI with a high chance of peri-procedural mortality.

Complex high-risk PCI can cause haemodynamic instability through a mechanism of procedure-induced ischaemia of the heart. This can cause acute heart failure and lead to a detrimental outcome of elective percutaneous interventions.

In the past, high-risk PCI could be performed under mechanical circulatory support using the intra-aortic balloon pump (IABP), but its use has seen a decline and downgrade in this regard as studies have failed to display a clear benefit of this form of mechanical circulatory support [6–8]. Recently, high-risk PCI under mechanical support, especially extracorporeal membrane oxygenation (ECMO) and the Impella device (Abiomed, Aachen, Germany), has been established as a possible alternative for this group of high-risk patients [9–12]. Initial reports on the use of veno-arterial (VA)-ECMO showed promising results in the treatment of cardiogenic shock due to myocardial infarction [13–15]. Patients with stable coronary artery disease have also been revascularised with mechanical support using VA-ECMO. However, data on outcome and procedural characteristics are scarce [9, 10].

The current study aims at describing patient characteristics, procedural findings and outcome in the use of VA-ECMO for mechanical circulatory support in elective high-risk PCI.

## Methods

A two-centre, retrospective study was performed, including all patients that underwent elective very high-risk PCI under ECMO for stable coronary artery disease in the past 18 months. All patients were analysed for age, gender, previous medical history including diabetes, hypertension, peripheral artery disease, tobacco use, renal function where an estimated glomerular filtration rate (eGFR) <60 (stage 3) was considered impaired renal function, and the presence of a malignant proliferative disease [16].

Patients were selected by the heart team and the attending interventional cardiologist, who also performed the ECMO-assisted PCI. In neither of the two participating centres was a form of mechanical circulatory support other than VA-ECMO available at the time that was capable of providing full mechanical circulatory support.

Cardiac status was analysed in regard to previous coronary artery disease and previous cardiac surgery, which was divided into previous CABG and valvular surgery. Left ventricular function was assessed using the available imaging modalities for each patient, including transthoracic echo and nuclear imaging when available. Left ventricular function was defined as good (>55%), mildly impaired (45–55%), moderately impaired (35–45%) or severely impaired (<35%) [17].

Procedural characteristics were analysed with regard to the number of target vessels that were revascularised (whereby all separate branches were counted as one separate vessel), achievement of complete revascularisation, duration of ECMO run in hours, limb ischaemia, post-procedural admission to the intensive care unit, thromboembolic complications, acute renal failure defined as an increase of >1 stage of renal failure above baseline, the need for haemodialysis, drop in haemoglobin post-procedure, drop in thrombocyte count post-procedure, neurological outcome (where a cerebral performance category (CPC) scale of 1 and 2 was deemed a good neurological outcome), major adverse cardiovascular events during admission, re-infarction and mortality at discharge [18].

For all patients SYNTAX scores I and II were calculated in order to assess coronary anatomy using the online calculator (http://www.syntaxscore.com/calculator/start.htm) and for all patients the J‑CTO score was also calculated when appropriate to assess lesion anatomy and complexity using the online J‑CTO score sheet (https://www.incathlab.com/files/COURSES/CTO/jcto-score-sheet.pdf). Furthermore, for all patients EuroSCORE I and II were calculated to assess the risk of peri-procedural mortality using the online calculator (http://www.euroscore.org/calc.html).

## Results

### Baseline clinical characteristics

Between January 2017 and August 2018, a total of 14 patients underwent high-risk PCI under prophylactic VA-ECMO support in two hospitals in the Netherlands. The majority were male (92%), with a median age of 69 (53–83) years, and 50% of all patients were over 70 years old. A previous history of hypertension was present in 57%, diabetes mellitus in 21%, documented hypercholesterolaemia in 29%, and 43% were known to have peripheral artery disease. No patient had an active malignancy at the time of this study. Renal function was impaired (eGFR <60, stage ≥3) in 21%. Median haemoglobin level prior to mechanical circulatory support was 8.2 (6.9–9.8) mmol/l and median thrombocyte count was 271 (166–594) $\times 10^{9}/\text{l}$ (see Tab. 1).

**Table 1:** Baseline characteristics (demographics, risk factors and biochemical values)

*Age (years)*	69 (53–83)
*Male gender*	92% (13/14)
*Diabetes mellitus*	21% (3/14)
*Hypercholesterolaemia*	29% (4/14)
*Hypertension*	57% (8/14)
*Peripheral arterial disease*	43% (6/14)
*Malignancy*	0% (0/14)
*Smoker*	29% (4/14)
*Impaired renal function (eGFR <60, stage ≥3)*	21% (3/14)
*Haemoglobin (mmol/l)*	8.2 (6.9–9.8)
*Thrombocytes (*$\times 10^{9}/\text{l}$*)*	271 (166–594)

*eGFR* estimated glomerular filtration rate

### Assessment of cardiac anatomy and function

Half of the patients had undergone previous coronary revascularisation in the form of CABG (36%) or PCI (14%). Left ventricular function was severely impaired (ejection fraction <35%) in 71% of the patients. In 21% the left ventricular ejection fraction was moderately impaired and in 7% mildly impaired. The main target vessel was the left main coronary artery in 71% of cases, left anterior descending artery in 21% and right coronary artery in 7%. Additional PCI of the non-target vessel was performed in 71%. The target vessel was a CTO in 79% of the lesions. Median J‑CTO score was 1 (0–3) (mean 1.55, SD 0.93). The median SYNTAX score I was 34 (8–42.5), the median SYNTAX score II (PCI) was 53.5 (26.2–79.5) and the median SYNTAX score II (CABG) was 40.1 (16.2–57.2) (see Tab. 2).

**Table 2:** Baseline characteristics (cardiac history, anatomy and risk scores)

Prior coronary artery disease	57% (8/14)
Prior CABG	36% (5/14)
Previous valvular surgery	0% (0/14)
Number of target vessels	3 (1–4)
LM	71% (10/14)
LAD	57% (8/14)
RCA	64% (9/14)
RCx	57% (8/14)
CTO	79% (11/14)
LVEF >55%	0% (0/14)
LVEF 45–55%	7% (1/14)
LVEF 35–45%	21% (3/14)
LVEF <35%	71% (10/14)
EuroSCORE I	7.1 (3.6–34.1)
EuroSCORE II	3.2 (0.9–16.8)
SYNTAX score I	34 (8–42.5)
SYNTAX score II (PCI)	53.5 (26.2–79.5)
SYNTAX score II (CABG)	40.1 (16.2–57.2)
J‑CTO score (median for all CTO lesions)	1 (0–3)

*CABG* coronary artery bypass graft; *LM* left main coronary artery; *LAD* left anterior descending artery; *RCA* right coronary artery; *RCx* ramus circumflexus; *CTO* chronic total occlusion; *LVEF* left ventricular ejection fraction; *PCI* percutaneous coronary intervention

### Procedural characteristics

Prior to cannulation all patients were intubated and put on mechanical ventilation. Cannulation was femoral-femoral in all patients. None of the patients received antegrade perfusion of the leg during the procedure. All patients were cannulated by the attending cardiothoracic surgeon. Median ECMO run was 2.57 h (1–4). In an open procedure all patients were decannulated at the catheterisation laboratory by the attending cardiothoracic surgeon without complications. None of the patients was admitted to the intensive care unit after the procedure. All patients were transferred to the coronary care unit without incident.

### Outcome

PCI was successful in 100% of the patients with revascularisation of all target and additional lesions. Survival to hospital discharge was 93%. One patient died during hospitalisation owing to refractory cardiac failure due to end-stage heart failure which was not attributed to either the PCI or the VA-ECMO support by the attending physician. All other patients were discharged neurologically intact with a CPC scale of 1. ECMO-related complications occurred in 14% of patients, with one patient developing a transient ischaemic attack after the procedure and one patient developing a thrombus in the femoral vein used for cannulation several days after decannulation. One patient was re-admitted after several months with a re-infarction. The median drop in haemoglobin was 2.0 (0.4–3.0) mmol/l and the median thrombocyte count drop was 82 (16–107) $\times 10^{9}/\text{l}$ (see Tab. 3).

**Table 3:** Procedural characteristics and outcomes

Complete revascularisation	100% (14/14)
Duration of ECMO (h)	3 (1–4)
Limb ischaemia	0% (0/14)
Post-procedural admission to ICU	0% (0/14)
Thromboembolic complication	14% (2/14)
Renal insufficiency post-procedure (increase ≥1 stage above baseline)	21% (3/14)
Need for haemodialysis	0% (0/14)
Haemoglobin drop (mmol/l)	2.0 (0.4–3.0)
*Thrombocyte drop (*$\times 10^{9}/\text{l}$*)*	82 (16–107)
MACE during admission	14% (2/14)
*Re-infarction*	7% (1/14)
*Mortality at discharge*	7% (1/14)

*ECMO* extracorporeal membrane oxygenation; *ICU* intensive care unit; *MACE* major adverse clinical events

## Discussion

The present study reports patient and procedural characteristics and short-term outcome in 14 patients who underwent high-risk PCI for stable coronary artery disease under prophylactic VA-ECMO support. Mortality and complications were low, and despite the highly complex and mostly extensive coronary artery disease of these patients PCI was successful in all cases.

PCI under ECMO in unstable patients with ST-elevation myocardial infarction and/or cardiogenic shock has been reported to be relatively safe with a good outcome [9, 13]. Literature about high-risk PCI under ECMO support in stable patients, however, is scarce to date. Tomasello et al. reported an excellent 6‑month outcome in a prospective study of 12 patients with stable coronary artery disease. However, complete revascularisation was accomplished in only 50% of these patients [19]. The present retrospective study shows a similar short-term outcome, but with complete revascularisation in all patients. This might be explained by operator experience. A large proportion of the patients had known CTO lesions, and it is in this specific group of patients that results are operator dependent. Highly skilled and experienced operators as well as clear revascularisation strategies increase the chance of success.

Decision-making about whether or not a specific patient requires haemodynamic support during high-risk PCI is not easy. Several factors have to be considered, such as left ventricular function, comorbidities, previous revascularisations, expected duration and complexity of PCI and expected ischaemic burden during PCI. For example, PCI of an unprotected left main coronary artery, especially when accompanied by significant right coronary artery disease—or even CTO, can lead to massive ischaemia. The expected duration of balloon inflation and the risk of complications such as dissection have to be estimated beforehand. Ostial left main coronary artery lesions might lead to a shorter duration of ischaemia than complex Medina 1,1,1 distal left main coronary artery lesions, for example. Coronary anatomy must be taken into account when selecting patients for ECMO-assisted revascularisation.

In the present study, patient characteristics and extent of coronary artery disease vary widely. This is reflected by the EuroSCORE I and II and SYNTAX scores I and II. This emphasises the fact that high-risk factors are partially incorporated in these risk stratification scores, but individual assessment and tailored decision-making remain of the utmost importance. The heart team plays a vital role in this respect.

Local expertise with VA-ECMO and availability of trained personnel are also key factors when applying this new technique. In the participating hospitals, the majority of patients have been referred from another hospital. This includes hospitals with cardiac interventional facilities but without expertise and availability of ECMO. In the future, increasing experience with this new strategy for haemodynamic support may improve the outcome and further reduce complications.

In prolonged ECMO therapy one of the major complications that can arise is leg ischaemia resulting from occlusion of the femoral artery due to the arterial cannula. None of the patients in this study underwent antegrade perfusion of the leg (so-called Leg-ECMO or L‑ECMO), yet none of the patients developed leg ischaemia. This underlines the safety of the procedure in respect to distal limb perfusion.

Use of femoral-femoral VA-ECMO increases afterload of the left ventricle, which is already being stressed by the PCI itself. This may cause a less optimal outcome. Simultaneous use of either the IABP or Impella device has been shown to improve the outcome in cardiogenic shock and may be considered to reduce afterload [20]. However, this does make the procedure more complex; the aorta is crowded with several devices, which may hamper catheter placement and cause femoral access to be unavailable for catheter insertion. It will also greatly increase the costs. In this study, ECMO alone has shown a good outcome and no form of afterload reduction was used. The short length of time required for ECMO as mechanical circulatory support may obviate the need for afterload reduction.

All patients were cannulated and decannulated by the attending surgeon. This may increase the safety of the procedure, but it will also stretch resources that could be otherwise put to good use. As experience with large-bore cannulas is increasing (e.g. transcatheter aortic valve implantation) and closure devices are progressively being used successfully (e.g. MANTA closure device, Teleflex Medical Europe Ltd, Athlone, Ireland; Perclose Proglide closure device, Abbott Vascular, Santa Clara, CA, USA) future procedures could be fully percutaneous with surgical expertise on-site. This may increase patient comfort, reduce scarring and also reduce the cost of the procedure.

In the application of VA-ECMO in the treatment of cardiogenic shock, regardless of its cause, age plays a major role in outcome. Patients over 70 are generally excluded from this form of mechanical circulatory support, as results in this group are quite poor with respect to outcome and complications [13]. In this study, however, half of the patients are over 70 years old, with some even over 80, and they do very well. This might be an indication that this revascularisation strategy with mechanical circulatory support is applicable to a much higher age group than is ECMO support for cardiogenic shock.

Besides prophylactic ECMO support, as was used in all patients in our present study, one might also consider provisional ECMO support. In this strategy, ECMO equipment and trained personnel will be readily available when needed, but only used when haemodynamic problems occur during the PCI procedure. An ECMO circuit will be present in the catheterisation laboratory, ready for use when needed. This might lead to fewer ECMO-related complications and reduce costs. However, on the downside, this strategy might lead to a delay in the treatment of cardiogenic shock and may increase poor outcome when cardiogenic shock occurs. Literature on provisional ECMO support is virtually absent as compared to prophylactic ECMO support. In the future, randomised trials will be necessary to answer this question and determine the best strategy for revascularisation with mechanical circulatory support.

As well as ECMO, other haemodynamic support systems are also available. These comprise Impella, IABP, TandemHeart (Cardiac Assist Inc., Pittsburgh, PA, USA) and PulsCath iVAC2L (PulseCath BV, Arnhem, The Netherlands). Of these devices, IABP results in a relatively low amount of mechanical circulatory support but showed no improvement in outcome in the BCIS‑1 trial [21]. In a prospective study by Kovacic et al., direct comparison in stable patients with three-vessel disease and impaired left ventricular function showed that the Impella device was associated with a significantly lower incidence of major adverse events in 90 days than IABP [11]. The advantage of Impella is that it is less invasive. The disadvantage is that it only provides flow but does not ensure end-organ perfusion. Furthermore, use of the Impella device might hamper catheter placement in the aorta. ECMO is more invasive but will ensure end-organ perfusion and not disturb catheter placement in the aorta. A direct comparison of prophylactic Impella and VA-ECMO support in high-risk PCI patients has not yet been performed to our knowledge. Local availability and expertise will largely guide the choice of which haemodynamic support device to use until there is a head-to-head comparison of these two techniques. Of the two other devices that have been used for mechanical support during high-risk PCI, the TandemHeart shows similar results to the Impella device [22], while the PulsCath device has demonstrated feasibility and safety in this group of patients, and first reports on outcome show promising results [23, 24]. A future head-to-head comparison between the different support modalities must show which of these is most suited in this group of patients.

## Conclusion

Our study shows good feasibility and good short-term outcome of prophylactic VA-ECMO-supported PCI in stable, high-risk patients. Mortality and complication rate are low. This procedure can be safely used to provide mechanical circulatory support in patients undergoing high-risk PCI. However, more prospective research and head-to-head comparison with other support devices is indicated to determine the best strategy in this vulnerable and high-risk group of patients.

## References

[1] Neumann FJ, Sousa-Uva M, Ahlsson A (2019). 2018 ESC/EACTS Guidelines on myocardial revascularization. Eur Heart J.

[2] Serruys PW, Morice MC, Kappetein AP (2009). Percutaneous coronary intervention versus coronary-artery bypass grafting for severe coronary artery disease. N Engl J Med.

[3] Mohr FW, Morice MC, Kappetein AP (2013). Coronary artery bypass graft surgery versus percutaneous coronary intervention in patients with three-vessel disease and left main coronary disease: 5-year follow-up of the randomised, clinical SYNTAX trial. Lancet.

[4] Roques F, Nashef SA, Michel P (1999). Risk factors and outcome in European cardiac surgery: analysis of the EuroSCORE multinational database of 19030 patients. Eur J Cardiothorac Surg.

[5] Windecker S, Kolh P, Alfonso F, Authors/Task Force (2014). 2014 ESC/EACTS Guidelines on myocardial revascularization: The Task Force on Myocardial Revascularization of the European Society of Cardiology (ESC) and the European Association for Cardio-Thoracic Surgery (EACTS). Developed with the special contribution of the European Association of Percutaneous Cardiovascular Interventions (EAPCI). Eur Heart J.

[6] Prondzinsky R, Lemm H, Swyter M (2010). Intra-aortic balloon counterpulsation in patients with acute myocardial infarction complicated by cardiogenic shock: the prospective, randomized IABP SHOCK Trial for attenuation of multiorgan dysfunction syndrome. Crit Care Med.

[7] Rihal CS, Naidu SS, Givertz MM (2015). 2015 SCAI/ACC/HFSA/STS clinical expert consensus statement on the use of percutaneous mechanical circulatory support devices in cardiovascular care (endorsed by the American Heart Association, the Cardiological Society of India, and Sociedad Latino Americana de Cardiologia Intervencion; affirmation of value by the Canadian Association of Interventional Cardiology-Association Canadienne de Cardiologie d’intervention). J Card Fail.

[8] Cheng JM, den Uil CA, Hoeks SE (2009). Percutaneous left ventricular assist devices vs. intra-aortic balloon pump counterpulsation for treatment of cardiogenic shock: a meta-analysis of controlled trials. Eur Heart J.

[9] Spiro J, Doshi SN (2014). Use of left ventricular support devices during acute coronary syndrome and percutaneous coronary intervention. Curr Cardiol Rep.

[10] Napp LC, Kuhn C, Hoeper MM (2016). Cannulation strategies for percutaneous extracorporeal membrane oxygenation in adults. Clin Res Cardiol.

[11] Kovacic JC, Kini A, Banerjee S (2015). Patients with 3-vessel coronary artery disease and impaired ventricular function undergoing PCI with Impella 2.5 hemodynamic support have improved 90-day outcomes compared to intra-aortic balloon pump: a sub-study of the PROTECT II trial. J Interv Cardiol.

[12] Lee WC, Fang CY, Chen HC (2016). Associations with 30-day survival following extracorporeal membrane oxygenation in patients with acute ST segment elevation myocardial infarction and profound cardiogenic shock. Heart Lung.

[13] van den Brink FS, Magan AD, Noordzij PG (2018). Veno-arterial extracorporeal membrane oxygenation in addition to primary PCI in patients presenting with ST-elevation myocardial infarction. Neth Heart J.

[14] Negi SI, Sokolovic M, Koifman E (2016). Contemporary use of veno-arterial extracorporeal membrane oxygenation for refractory cardiogenic shock in acute coronary syndrome. J Invasive Cardiol.

[15] Agarwal S, Sud K, Martin JM, Menon V (2015). Trends in the use of mechanical circulatory support devices in patients presenting with ST-segment elevation myocardial infarction. JACC Cardiovasc Interv.

[16] Levey A, de Jong P, Coresh J (2011). The definition, classification, and prognosis of chronic kidney disease: a KDIGO Controversies Conference report. Kidney Int.

[17] Lang RM, Bierig M, Devereux RB (2006). Recommendations for chamber quantification. Eur J Echocardiogr.

[18] Levy DE, Caronna JJ, Singer BH, Lapinski RH, Frydman H, Plum F (1985). Predicting outcome from hypoxic-ischemic coma. JAMA.

[19] Tomasello SD, Boukhris M, Ganyukov V (2015). Outcome of extracorporeal membrane oxygenation support for complex high-risk elective percutaneous coronary interventions: a single-center experience. Heart Lung.

[20] Russo JJ, Aleksova N, Pitcher I (2019). Left ventricular unloading during extracorporeal membrane oxygenation in patients with cardiogenic shock. J Am Coll Cardiol.

[21] Perera D, Stables R, Clayton T (2013). Long-term mortality data from the balloon pump-assisted coronary intervention study (BCIS-1): a randomized, controlled trial of elective balloon counter pulsation during high-risk percutaneous coronary intervention. Circulation.

[22] Briasoulis A, Telila T, Palla M (2016). Meta-analysis of usefulness of percutaneous left ventricular assist devices for high-risk percutaneous coronary interventions. Am J Cardiol.

[23] den Uil CA, Daemen J, Lenzen MJ (2017). Pulsatile iVAC 2L circulatory support in high-risk percutaneous coronary intervention. EuroIntervention.

[24] Ameloot K, Bastos M, Daemen J (2019). New generation mechanical circulatory support during high-risk PCI: a cross sectional analysis. EuroIntervention.

